# Large-Scale Multicast Group Secure Transmission Scheme Based on Multi-Carrier FDA

**DOI:** 10.3390/s23239358

**Published:** 2023-11-23

**Authors:** Dan Wu, Yuqi Fei, Jianbang Gao, Fei Wang, Guowang Gao

**Affiliations:** School of Electronic Engineering, Xi’an Shiyou University, Xi’an 710065, China; wudan@xsyu.edu.cn (D.W.); 21212030397@stumail.xsyu.edu.cn (Y.F.); gjbang2023@xsyu.edu.cn (J.G.); 200102@xsyu.edu.cn (F.W.)

**Keywords:** physical layer security, large-scale multicast group, layered transmission, artificial noise

## Abstract

Aiming at the problem that the traditional physical layer technology cannot realize secure transmission due to the large number of users and wide dispersion in the multicast system, a layered transmission method is proposed, and a scheme for the secure transmission of the multicast group physical layer is designed. Firstly, the hierarchical transmission system model is established. Then, the array weighted vector of each layer is optimized according to the design criterion of maximizing the artificial noise interference power. At the same time, in the case where the number of users in a single multicast group is greater than the number of transmitting antennas, a multicast grouping strategy is introduced, and the singular value decomposition and Lagrange multiplier algorithms are utilized to obtain the optimal solution. Simulation results show that the proposed method can realize the secure communication of users with different distances in the same direction and can distinguish the multicast users with the same direction angle and different distances under the premise of mutual non-interference, thus realizing the secure communication of large-scale multicast users.

## 1. Introduction

With the advancement of mobile communication technology, wireless communication has been widely used in military and civilian fields, and new wireless systems such as the Internet of Things [1], Unmanned Aerial Vehicle(UAV) communication [2], and vehicular networks [3] have also been developed vigorously. However, due to the openness of wireless channels, the transmitted information is easily intercepted or eavesdropped on by eavesdroppers [4,5,6]. It is especially important to ensure the security of information in today’s world, where everything is interconnected, and the amount of data in wireless communication is increasing. Information security will become the focus and difficulty of wireless communication development. Traditional wireless communication mainly relies on high-level encryption technology to ensure the security of information [7]. With the increase in mobile devices and the emergence of supercomputers, the problem of information leakage in wireless communication relying on high-level encryption technology has emerged continuously. Information security faces increasing challenges.

Unlike traditional encryption techniques, physical layer security techniques [8] utilize the inherent properties of the wireless channel to provide more basic security for wireless communications. Array antenna [9] is an emerging multi-antenna physical layer security technique that utilizes the difference between the desired channel and the eavesdropping channel to achieve secure transmission of information [10,11]. Currently, there are still problems in the theoretical research and practical application of array antenna physical layer security technology that need to be urgently addressed. In practical communication scenarios, eavesdroppers often passively receive information with a certain degree of concealment. In actual communication scenarios, eavesdroppers often receive information passively, with a certain degree of concealment. The transmitter cannot accurately obtain the eavesdropper’s location information, thus reducing the security performance of the system. At the same time, in previous studies, mostly the physical layer secure communication problems of single-expectant-user communication systems or multi-expectant-user broadcast communication systems have been studied, and there have been fewer studies on complex multicast group communication systems. For example, Qiu et al. investigated the secure transmission problem of broadcast secure communication systems with multiple desired targets and a single eavesdropper [12]. Gao et al. solved the physical layer secure communication problem with a single legitimate target when the eavesdropper’s location is unknown by using the method of maximizing artificial noise [13]. If the secure unicast communication technique is directly applied to multicast communication, it will not be able to add effective artificial noise in the orthogonal space of the legitimate channel vectors, thus not guaranteeing secure data transmission. Secure communication schemes in the literature [13,14] are mainly based on the principle of maximizing the secrecy capacity and studying the secure multicast group communication problem when the eavesdropper is unknown. The literature [15] analyzes a multicast secure communication technique using a two-dimensional beam assignment combined with artificial noise. Some literature [16] has investigated non-orthogonal multiple access (NOMA) assisted secure offloading for vehicular edge computing (VEC) networks in the presence of multiple malicious eavesdroppers. The Physical Layer Security (PLS) technique is employed to provide jamming signals to eavesdroppers without interfering with real users. Danyu Diao et al. [17] proposed a joint power allocation and air jamming (PAAJ) scheme to achieve reliable and secure communication of the system in the presence of malicious eavesdroppers. Bingcai Chen et al. used multiple antenna relays to enhance the communication and to obtain the diversity gain [18]. The above literature research methods are limited by the number of multicast users and the number of eavesdroppers. In scenarios where a large number of multicast users and eavesdroppers coexist, traditional physical layer security techniques cannot solve the problem of secure transmission of large-scale multicast groups well. However, the research on physical layer large-scale multicast group security transmission technology is of great academic research value and practical guidance significance for supporting the communication demand of 6G large-scale devices and realizing the significant improvement of 6G communication network security performance.

Multicast group communication [19] can provide large-capacity and diverse data services for a large number of devices, which makes it suitable for large-scale wireless communication systems. Therefore, multicast group communication is characterized by a large number of desired users and a wide range of spatial dispersion, and the traditional directional modulation technique cannot realize the secure communication of users with different distances in the same direction. In addition, the artificial noise scrambling technique mainly lies in constructing the zero space of the desired channel, which is limited by the number of transmitting antennas (the number of transmitting antennas is larger than the number of desired users). Traditional physical layer security techniques cannot perfectly guarantee the security of multicast systems, and more dimensional redundancy needs to be introduced to solve such problems. Based on this, the main contributions of this paper are:

1. A layered transmission method is proposed, and the transmitter transmits the confidential information in layers while an artificial noise matrix is designed to ensure transmission efficiency.
2. User grouping strategy is added to the traditional method, and a multicast user grouping multi-carrier frequency controlled array secure transmission scheme is proposed.

## 2. System Model

In this section, firstly, the model schematic of the multicast group system is introduced, and the structure of the multi-carrier frequency controlled array (MFCA) transmitter is analyzed; then, the theoretical derivation and a secure transmission scheme based on the MFCA in the physical layer is proposed.

**Figure 1:**
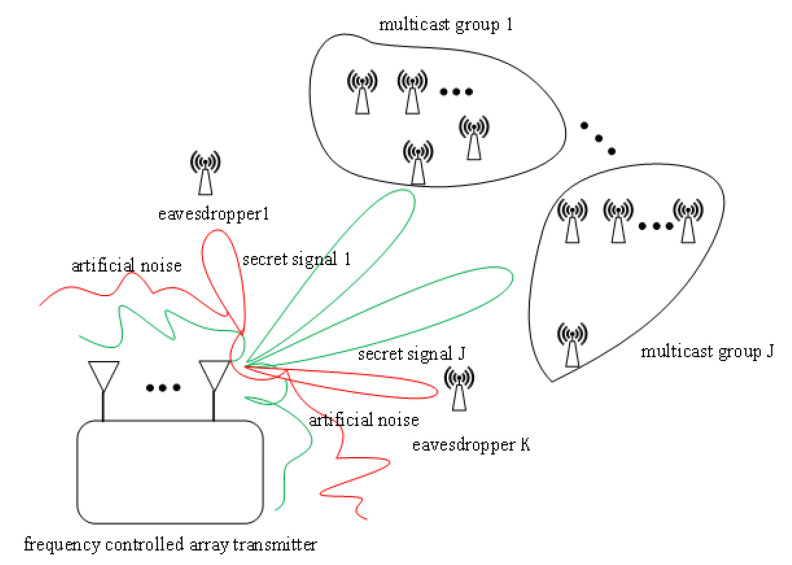
Multicast group system model.

Consider the basic multicast system as shown in Figure 1. The system contains a transmitting base station and *J* multicast groups while assuming the presence of one or more passively receiving eavesdroppers around each multicast group. Users within the same multicast group receive the same multicast message, and users within the same multicast group can be spatially dispersed. Assume that the *j*th multicast group contains $G_{j}$ legitimate stations.

**Figure 2:**
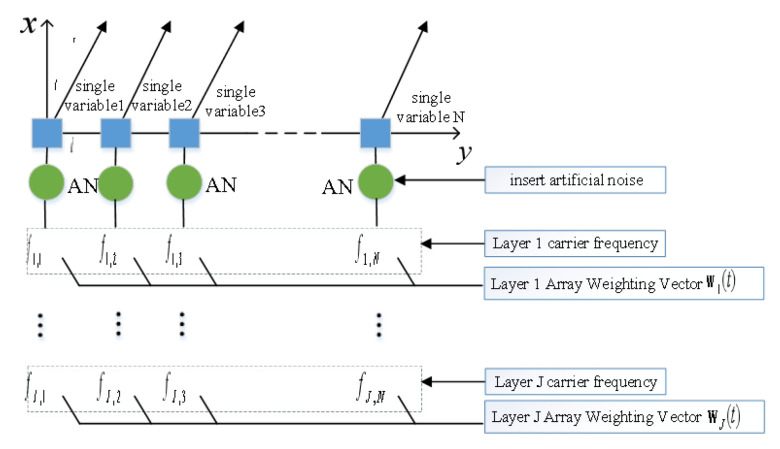
Multi-carrier frequency-controlled array transmitter structure.

The number of expected users in multicast group communication systems is large, and the traditional physical layer secure transmission technique cannot perfectly guarantee the communication security of multicast systems. Therefore, a physical layer secure transmission scheme based on a multi-carrier frequency-controlled array is proposed. First, the multi-carrier frequency-controlled array transmitter is designed, as shown in Figure 2. The frequency-controlled array antenna consists of *N* equally spaced omni-directional antennas linearly distributed, assuming that the spacing between neighboring antennas is *d*. The position of the first transmitter antenna is determined to be the origin of the system coordinates, and the effect of multipath transmission is ignored. Each transmitting antenna in the multi-carrier frequency-controlled array transmitter no longer transmits the traditional single-carrier signal but transmits multi-carrier signals with different frequencies.

As shown in Figure 2, the number of subcarriers of each antenna in the multi-carrier frequency-controlled array transmitter is *J*. The subcarriers of the same order of each antenna are defined as a layer.

The subcarrier of the *n*th transmitting antenna in the *j*th layer is denoted as

(1) $$f_{j,n}=f_{j,c}+\Delta f_{j,n},j=1,2,\cdots ,J,n=1,2,\cdots ,N$$

where $f_{j,c}$ is the carrier frequency of the *j*th layer; $\Delta f_{j,n}$ is the frequency offset between different antennas in the *j*th layer.

The energy of the radiated signal from the *j*th layer received by the far-field target (r,θ) is expressed as

(2) $$\begin{array}{cc} y_{j}\left(r,\theta \right) & =\sum_{n=1}^{N}\exp\{j2\pi f_{j,n}\left(t-\frac{r_{n}}{c}\right)\}\approx \exp\{j2\pi f_{j,c}\left(t-\frac{r}{c}\right)\}\\ & \times \sum_{n=1}^{N}\exp\{j\left[2\pi \Delta f_{j,n}\left(t-\frac{r}{c}\right)+\frac{2\pi f_{j,c}nd\sin\theta }{c}\right]\} \end{array}$$

where *r* denotes the distance from the coordinate origin far-field target user; $r_{n}$ denotes the distance from the *n*th antenna to the target user; *c* denotes the speed of light.

Then, for the far-field target (r,θ), the *j*th layer array guidance vector is written as

(3) $$\begin{array}{c} h_{L,j}=\frac{1}{\sqrt{N}}[e^{j(\Delta f_{j,1}\left(t-\frac{r}{c}\right)+\frac{2\pi f_{j,c}nd\sin\theta }{c})},\\ \cdots ,e^{j(\Delta f_{j,n}\left(t-\frac{r}{c}\right)+\frac{2\pi f_{j,c}nd\sin\theta }{c})},\\ \cdots ,e^{j(\Delta f_{j,N}\left(t-\frac{r}{c}\right)+\frac{2\pi f_{j,c}nd\sin\theta }{c})}{]}^{T} \end{array}$$

Thus, the energy of all layers transmitted signals at the far-field target point is expressed as

(4) $$\begin{array}{c} y\left(r,\theta \right)\approx \sum_{j=1}^{J}\sum_{n=1}^{N}\exp\{j2\pi f_{j,c}\left(t-\frac{r}{c}\right)\}\\ \exp\{j\left[2\pi \Delta f_{j,n}\left(t-\frac{r}{c}\right)+\frac{2\pi f_{j,c}nd\sin\theta }{c}\right] \end{array}$$

Then, the guidance vector of the multi-carrier array is

(5) $$h_{L}=\frac{1}{\sqrt{NJ}}{\left[h_{L,1}^{T}\cdots h_{L,j}^{T}\cdots h_{L,J}^{T}\right]}^{T}$$

In this case $h_{L}\in C^{NJ\times 1}$ is the NJ dimensional vector. The application scale of the multicast system is expanded without increasing the transmitting antenna.

## 3. Multi-Carrier Massive Multicast Group Secure Communication Methods

In this section, based on the multi-carrier frequency controlled array transmitter, the layered transmission method is proposed, and based on this, the multi-carrier large-scale multicast group secure communication method is designed.

The number of available carriers per transmitter antenna is *J*; that is, the system is divided into *J* layers for transmission. First, the users receiving the same information are divided into the same multicast group, while the number of users within this multicast group is less than the number of antennas at the transmitter. Second, the transmitter transmits the confidential information in layers, and the *j*th layer transmits the modulation symbol $x_{j}\left(t\right)$, which corresponds to all the users within the multicast group *j*. The layered design of the array weighting vector $w_{j}$ ensures that the users within multicast group *j* receive the signal normally without interfering with other users within the multicast group. Subsequently, artificial noise is introduced before each antenna radiates the signal, and the artificial noise matrix is also designed so that the artificial noise is transmitted in the zero space of the desired channel, which interferes with the eavesdroppers while not affecting the users. Finally, at the receiving end, the multicast group selects the appropriate subcarrier to demodulate the received signal.

The transmit signal vector $s\left(t\right)=[s_{1}\left(t\right),s_{2}\left(t\right),\cdots ,s_{N}\left(t\right)]$ at moment *t*, denoted as

(6) $$s\left(t\right)=\sum_{j=1}^{J}w_{j}x_{j}\left(t\right)+\sqrt{P_{AN}}v_{AN}\left(t\right)$$

where: $w_{j}$ represents the *j*th-layer array weighted vector; $P_{AN}$ stands for artificial noise emission power; $w_{j}\left(t\right)\overset{\Delta }{=}A_{j}\left(t\right)b_{j}\left(t\right)$ where $\begin{array}{c} \min_{b_{j}\left(t\right)}b_{j}^{H}(t)A_{j}^{H}(t)A_{j}(t)b_{j}(t)\\ s.t.H_{L,j_{j}}^{H}\left(t\right)A_{j}(t)b_{j}(t)\geq \xi_{j} \end{array}$ denotes the artificial noise orthogonal projection matrix; *j* denotes the artificial noise vector that follows a normal distribution, i.e., $\begin{array}{c} w_{j}^{*}\left(t\right)=A_{j}\left(t\right)(A_{j}^{H}\left(t\right)A_{j}\left(t\right){)}^{-1}A_{j}^{H}\left(t\right)H_{L,j_{j}}\left(t\right)\\ {\left[H_{L,j_{j}}^{H}\left(t\right)A_{j}\left(t\right)(A_{j}^{H}\left(t\right)A_{j}\left(t\right){)}^{-1}A_{j}^{H}\left(t\right)H_{L,j_{j}}\left(t\right)\right]}^{-1}\xi_{{}_{j}}^{H} \end{array}$.

Assume that the location of the-*g*th user within multicast group *j* is $\left(r_{L,j_{g}},\theta_{L,j_{g}}\right)$, The user’s guiding vector is $h_{L,j_{g}}={\left[h_{L,j_{g,1}}^{T}\cdots h_{L,j_{g,j}}^{T}\cdots h_{L,j_{g,J}}^{T}\right]}^{T}$. With this, the co-direction matrix of users within a multicast group *j* is denoted as

(7) $$H_{L,j}\left(t\right)\overset{\Delta }{=}\left[h_{L,j_{1}}\left(t\right),h_{L,j_{2}}\left(t\right),\cdots ,h_{L,j_{G_{j}}}\left(t\right)\right]$$

The layer *j* orientation matrix for a user within multicast group *j* is denoted as

(8) $$H_{L,j_{j}}\left(t\right)\overset{\Delta }{=}\left[h_{L,j_{1,j}}\left(t\right),h_{L,j_{2,j}}\left(t\right),\cdots ,h_{L,j_{G_{j,j}}}\left(t\right)\right]$$

The received signal vector of multicast group *j* is represented as

(9) $$y_{L,j}\left(t\right)=H_{L,j_{j}}^{H}\left(t\right)w_{j}\left(t\right)x_{j}\left(t\right)+\sum_{i=1,i\neq j}^{J}H_{L,j_{i}}^{H}\left(t\right)w_{i}\left(t\right)x_{i}\left(t\right)+\sqrt{P_{AN}}H_{L,j}^{H}\left(t\right)v_{AN}\left(t\right)+n_{L,j}\left(t\right)$$

where: $n_{L,j}\left(t\right)∼CN\left(0,\sigma_{L,j}^{2}I_{G_{j}}\right)$ denotes the additive Gaussian white noise matrix of the multicast group *j*.

Observing Equation (9), it can be found that the received signal vector of multicast group *j* consists of four parts. The first part is the modulated symbol $x_{j}\left(t\right)$ received by multicast group *j* transmitted by layer *j*; the second part is the interference signal transmitted by other layers; the third part is the artificial noise; and the fourth part is the channel noise.

First, the layer *j* array weighting vector $w_{j}$ is designed with the design criterion of ensuring that users within a multicast group *j* receive signals normally while not interfering with users within other multicast groups. One or more eavesdroppers exist around or inside each multicast group to steal information, and it is often impossible to obtain the information of eavesdroppers in actual wireless communication. At this time, it mainly relies on artificial noise to interfere with the eavesdropper so that it can not properly demodulate the confidential information. Therefore, in the case of ensuring that the user receives the signal normally maximizes the artificial noise transmit power, the optimization problem is described as

(10) $$\begin{array}{c} \max_{w_{j}\left(t\right)}P_{AN}\\ s.t.H_{L,j_{j}}^{H}\left(t\right)w_{j}\left(t\right)\geq \xi_{j}\\ H_{L,-j_{j}}^{H}w_{j}\left(t\right)=0_{L_{-j}\times 1} \end{array}$$

where $H_{L,-j_{j}}\left(t\right)\overset{\Delta }{=}\left[H_{L,1_{j}}\left(t\right),\cdots ,H_{L,j-1_{j}}\left(t\right),H_{L,j+1_{j}}\left(t\right),\cdots ,H_{L,J_{j}}\left(t\right)\right]$ denotes the layer *j* orientation matrix of the remaining multicast group *j* outside the multicast group. $\xi_{j}\overset{\Delta }{=}{\left[\sqrt{\xi_{j,1}},\sqrt{\xi_{j,2}},\cdots ,\sqrt{\xi_{j,g}},\cdots \sqrt{\xi_{j,G_{j}}}\right]}^{T}$, $\xi_{j,g}$ indicates the minimum receive power required by users in a multicast group. $L_{-j}=\sum_{i=1,i\neq j}^{J}G_{i}$ denotes the number of users remaining outside of multicast group *j*. Constraint 1 indicates that all users in the multicast group *j* receive the transmission signals of layer *j* and meet the minimum receive power requirement. Constraint 2 indicates that users outside the multicast group *j* are unable to receive the transmission signals of layer *j*. The total system transmit power is $P_{s}=P_{AN}+\sum_{j=1}^{J}P_{L,j}$, where $P_{L,j}$ represent the transmitted signal power of layer *j*. It is easy to find that the transmitting power of artificial noise can be maximized by means of the method of minimum modulation symbol transmitting power, and the optimization problem can be rewritten as

(11) $$\begin{array}{c} \min_{w_{j}\left(t\right)}{∥w_{j}\left(t\right)∥}_{2}^{2}\\ s.t.H_{L,j_{j}}^{H}\left(t\right)w_{j}\left(t\right)\geq \xi_{j}\\ H_{L,-j_{j}}^{H}w_{j}\left(t\right)=0_{L_{-j}\times 1} \end{array}$$

To solve this optimization problem, a singular value decomposition of the matrix $H_{L,-j_{j}}^{H}$ yields

(12) $$\begin{array}{c} H_{L,-j_{j}}^{H}=\left[U_{L,-j_{j}}^{\left(1\right)}\left(t\right)U_{L,-j_{j}}^{\left(0\right)}\left(t\right)\right]\left[\begin{array}{c} \sum_{L,-j_{j}}^{\left(1\right)}\left(t\right)0\\ 00 \end{array}\right]{\left[V_{L,-j_{j}}^{\left(1\right)}\left(t\right)V_{L,-j_{j}}^{\left(0\right)}\left(t\right)\right]}^{H} \end{array}$$

According to the singular value decomposition theorem, we can get $H_{L,-j_{j}}^{H}\left(t\right)v_{i}\left(t\right)=0$, where $v_{i}\in V_{L,-j_{j}}^{\left(0\right)}\left(t\right)$, $i=1,2,\cdots ,N\left(J-1\right)-L_{-j}$. Assuming that $A_{j}\left(t\right)\overset{\Delta }{=}V_{L,-j_{j}}^{\left(0\right)}\left(t\right)$ and $w_{j}\left(t\right)\overset{\Delta }{=}A_{j}\left(t\right)b_{j}\left(t\right)$, at this point Equation (12) is converted to

(13) $$\begin{array}{c} \min_{b_{j}\left(t\right)}b_{j}^{H}(t)A_{j}^{H}(t)A_{j}(t)b_{j}(t)\\ s.t.H_{L,j_{j}}^{H}\left(t\right)A_{j}(t)b_{j}(t)\geq \xi_{j} \end{array}$$

According to the Lagrange multiplier method, the *j*th layer optimal array weighted vector is obtained

(14) $$\begin{array}{c} w_{j}^{*}\left(t\right)=A_{j}\left(t\right)(A_{j}^{H}\left(t\right)A_{j}\left(t\right){)}^{-1}A_{j}^{H}\left(t\right)H_{L,j_{j}}\left(t\right)\\ {\left[H_{L,j_{j}}^{H}\left(t\right)A_{j}\left(t\right)(A_{j}^{H}\left(t\right)A_{j}\left(t\right){)}^{-1}A_{j}^{H}\left(t\right)H_{L,j_{j}}\left(t\right)\right]}^{-1}\xi_{{}_{j}}^{H} \end{array}$$

Next, the artificial noise matrix is calculated. According to the criterion that artificial noise interferes with the eavesdropper without affecting the signal received by the user, the artificial noise matrix is calculated by the following equation

(15) $$\mathrm{tr}\{T_{AN}^{H}\left(t\right)H_{L}\left(t\right)H_{L}^{H}\left(t\right)T_{AN}\left(t\right)\}=0$$

where: tr{•} denotes the trace of the matrix; $H_{L}\left(t\right)\overset{\Delta }{=}\left[H_{L,1}\left(t\right),\cdots ,H_{L,j}\left(t\right),\cdots ,H_{L,J}\left(t\right)\right]$ represents the oriented vector matrix of all multicast groups.

It is easy to find that the multicast group’s guiding vector matrix $H_{L}\left(t\right)\in C^{NJ\times L_{T}}$, where $L_{T}=\sum_{i=1}^{J}G_{i}$ indicates the number of users in all multicast groups. According to the null space mapping criterion, the artificial noise matrix can be calculated when $NJ>L_{T}$

(16) $$T_{AN}\left(t\right)=I_{NJ}-H_{L}\left(t\right){\left[H_{L}^{H}\left(t\right)H_{L}\left(t\right)\right]}^{-1}H_{L}^{H}\left(t\right)$$

The traditional artificial noise technology is based on the single carrier transmitting antenna array. At this time, the number of transmitting antennas must be greater than the number of multicast users; that is, $N>L_{T}$ can realize the interference with the eavesdropper without affecting the user’s receiving signal. However, the artificial noise jamming technology based on a multi-carrier frequency array can still realize the interference of eavesdroppers when the number of users is expected to be $N<L_{T}<NJ$ without affecting the received signal of users. Without increasing the transmission antenna, the scale of the multicast system is expanded to ensure more users communicate securely.

## 4. Solution Extension

In this section, based on the theoretical analysis in the previous two sections, the communication problem when the number of multicast groups and the number of carriers are unequal is further discussed in depth, and a solution is given for the problem, which proposes a secure transmission scheme for multicast user groups with multi-carrier frequency-controlled arrays.

The multi-carrier large-scale multicast group secure transmission scheme proposed above is carried out under the assumption that the number of multicast groups and the number of multi-carriers are equal, and the multicast secure communication problem should be analyzed further when the number of multicast groups and the number of carriers are not equal. It is assumed that the number of multicast groups is *Q* and the number of carriers in the transmitting antenna array is *J*.

When the number of carriers in the transmitting antenna array is greater than the number of multicast groups, that is, J>Q, *Q* carriers can be randomly selected for secure multicast group communication. In this case, there are a total of $C_{Q}^{J}$ options. Rational use of the randomness of transmitted carriers can improve the security of multicast group communication. However, the number of carriers in the antenna array *J* is greater than the number of multicast groups *Q*, and the number of users in the multicast group *q* is greater than the number of transmitting antennas, that is, $G_{q}>N$. In this case, secure communication in the multicast group *q* cannot be guaranteed. Aiming at the problem of secure communication in such scenarios, we continue to improve the proposed scheme by adding the user grouping strategy to the original method and propose a multicast user grouping scheme for secure transmission of multi-carrier frequency-controlled arrays.

When the number of users in the multicast group *q* is greater than the number of transmitting antennas, a user grouping strategy is introduced to regroup the users in the multicast group *q*. The basic principle is to divide the users whose guidance vector approaches into the same group, which is convenient for the transmitter to optimize the array weighted vector. The number of multicast group *q* packets is limited to $\left\lceil G_{q}/N\right\rceil$, where $\left\lceil •\right\rceil$ indicates that the multicast group is rounded up and smaller than J−Q. Under this limit, the number of users in each group can be ensured to be less than the number of transmitter antennas, and the number of carriers in the transmitting antenna array can be reduced to avoid the waste of wireless spectrum resources. Define the average channel similarity of expected user *i* as

(17) $$a_{i,j}\overset{\Delta }{=}\frac{1}{G_{q}}\sum_{j=1,j\neq i}^{G_{q}}\frac{\left|h_{L,i}^{H}\left(t\right)h_{L,j}\left(t\right)\right|}{∥h_{L,i}\left(t\right)∥∥h_{L,j}\left(t\right)∥}$$

The user grouping strategy consists of two phases, i.e., the group head selection phase and the grouping phase. In the group head selection phase, the average channel similarity of each user is first calculated and subsequently ranked, and the top $\left\lceil G_{q}/N\right\rceil$ users are selected as group heads. In the grouping phase, the remaining users are grouped together with the group heads with similar guidance vectors.

When the number of transmitter array antenna carriers is less than the number of multicast groups, that is, J<Q, and multicast user $L_{T}<NJ$, secure communication in the multicast system can only be achieved by continuing to increase the number of carriers at the transmitter side. At this time, the number of users in each multicast group is less, which can achieve a larger security capacity but increases the consumption of wireless resources and lower spectrum utilization.

## 5. Security Performance Analysis

In order to evaluate the security level of wireless communication systems, the security capacity is the main technical indicator. In this section, the proposed method is evaluated for security using security capacity as a criterion.

The Signal-to-Interference-plus-Noise Ratio (SINR) of the multicast group is expressed as

(18) $$\begin{array}{c} \gamma_{j}^{L}=\\ \frac{\mathrm{tr}[H_{L,j_{j}}^{H}\left(t\right)w_{k}\left(t\right)w_{k}^{H}\left(t\right)H_{L,j_{j}}\left(t\right)]/G_{j}}{\left\{\sum_{i=1,i\neq j}^{J}\mathrm{tr}[H_{L,j_{i}}^{H}\left(t\right)w_{i}\left(t\right)w_{i}^{H}\left(t\right)H_{L,j_{i}}\left(t\right)]\right\}/L_{-j}+P_{AN}\mathrm{tr}[H_{L,j}^{H}\left(t\right)n_{AN}\left(t\right)n_{AN}^{H}\left(t\right)H_{L,j}\left(t\right)]/G_{j}+\sigma_{L,j}^{2}} \end{array}$$

According to Equation (18), the maximum reachable rate of multicast group *j* is obtained

(19) $$C_{j}^{L}=\log_{2}\left(1+\gamma_{j}^{L}\right)$$

For multicast group *j*, the receiver SINR of the eavesdropper is

(20) $$\begin{array}{c} \gamma_{j,k}^{E}=\max_{\left(r_{E},\theta_{E}\right)\in S_{\mathrm{wire}}}\left(1+\frac{h_{E}^{H}\left(t\right)w_{j}\left(t\right)w_{j}^{H}\left(t\right)h_{E}\left(t\right)}{\sum_{i=1,i\neq j}^{J}h_{E}^{H}\left(t\right)w_{j}\left(t\right)w_{j}^{H}\left(t\right)h_{E}\left(t\right)+P_{AN}h_{E}^{H}\left(t\right)v_{AN}\left(t\right)v_{AN}^{H}\left(t\right)h_{E}\left(t\right)+\sigma_{L,j}^{2}}\right) \end{array}$$

According to Equation (20), the maximum rate that can be achieved from transmitter to eavesdropper is obtained

(21) $$C_{j,k}^{L}=\log_{2}\left(1+\max_{\left(r_{E},\theta_{E}\right)\in S_{wire}}\gamma_{j,k}^{E}\right)$$

Combined with Equations (19) and (21), the security capacity of multicast group *j* in the proposed scheme is expressed as

(22) $$C_{j}={\left[C_{j}^{L}-C_{j,k}^{E}\right]}^{+}$$

At the same time, the average safe capacity of the system is expressed as

(23) $$C_{s}=\frac{1}{J}\sum_{i=1}^{J}{\left[C_{i}^{L}-C_{i,k}^{E}\right]}^{+}$$

## 6. Simulation Results and Analysis

In this section, the distribution of SINR under different conditions is comprehensively analyzed based on the artificial noise interference power. Finally, comparing the method proposed in this paper with the traditional phased array multicast group secure transmission method, it is proved that the method in this paper can realize the secure communication of users with different distances in the same direction and, at the same time, it can distinguish the multicast users with different distances in the same direction angle.

The simulation parameters are set as follows: the number of frequency-controlled array antennas N=8. The carrier frequency of layer1 of the antenna array is $f_{1,c}=$ 1 GHz, and the carrier frequency of layer2 is $f_{2,c}=2$ GHz. The minimum safe receiving power for all multicast users is −90 dBm. Assuming that the number of multicast groups J=2, in the multicast system and the number of users in each multicast group, is $G_{1}=3,\;G_{2}=3$, and the user position of multicast group1 is $\left(r_{1,1},\;\theta_{1,1}\right)=(1200$ m, 30°), $\left(r_{1,2},\;\theta_{1,2}\right)=(1400$ m, 40°), $\left(r_{1,3},\;\theta_{1,3}\right)=(1600$ m, 35°) of multicast group1. User position of multicast group2 is $\left(r_{2,1},\;\theta_{2,1}\right)=(3200$ m, 40°), $\left(r_{2,2},\;\theta_{2,2}\right)=(3400$ m, 60°), $\left(r_{2,3},\;\theta_{2,3}\right)=(3600$ m, 50°).

**Figure 3:**
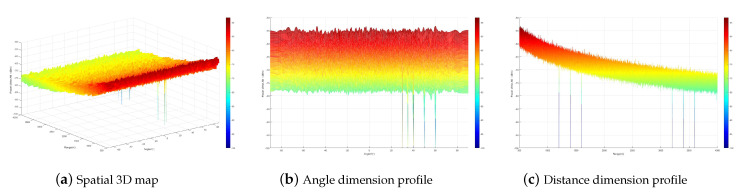
Artificial noise interference power distribution of multicast groups.

**Figure 4:**
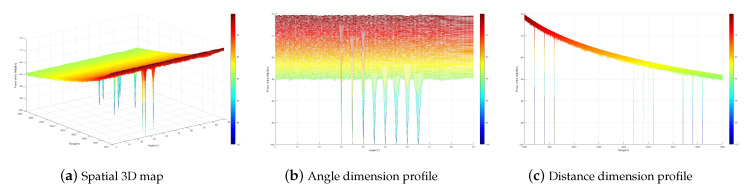
Artificial noise interference power distribution when the number of multicast users is 9.

Figure 3 simulates the artificial noise interference power distribution. From the figure, it can be seen that the artificial noise interference power forms a zero-sag at the multicast user location while it is uniformly distributed in the rest of the space. This indicates that when the multicast users are less than the transmitting antennas, both the single-carrier secure communication method and the multi-carrier secure transmission method can be designed to realize that the artificial noise does not affect the multicast users while interfering with the eavesdroppers by designing the artificial noise matrix. When the number of multicast users is $L_{T}=9$, the artificial noise interference power distribution of the multi-carrier secure transmission method is shown in Figure 4. From the figure, it can be seen that the artificial noise interference power forms zero traps at all multicast user locations. This indicates that the artificial noise technique based on a multi-carrier frequency-controlled array can ensure secure communication for more users without increasing the transmission antenna.

**Figure 5:**
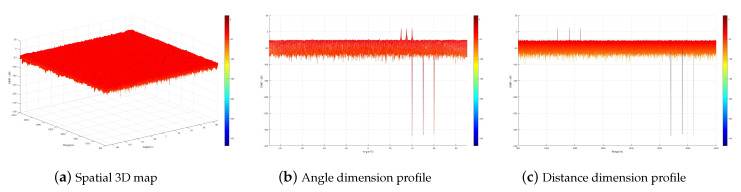
Received SINR distribution for multicast group1.

**Figure 6:**
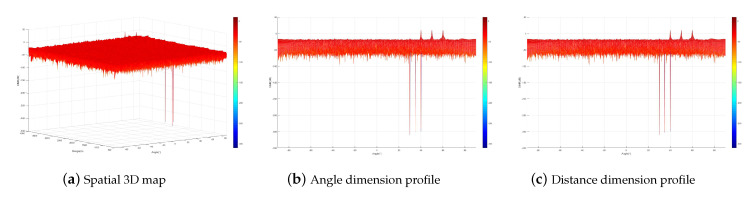
Received SINR distribution for multicast group2.

Subsequently, the received SINR distribution of the multicast group is analyzed. Figure 5 corresponds to multicast group1 and Figure 6 corresponds to multicast group2. Analyzing Figure 5, it is found that the peak SINR occurs at the user location of multicast group1 and is minimal at the user location of multicast group2. In addition to this, SINR is also very small at other locations. Analyzing Figure 6, a similar situation with multicast group1 can be obtained, which shows that after the optimization of the proposed scheme, the multicast group effectively receives the corresponding confidential signals, and the other multicast groups are unable to receive the signals that are not their own signals, and the eavesdroppers are even more unable to steal any confidential information. At the same time, compared with the traditional phased-array multicast group secure transmission method, the proposed method realizes the secure communication of users with different distances in the same direction and is able to distinguish multicast users with the same direction angle and different distances that are user $\left(r_{1,2},\;\theta_{1,2}\right)=(1400$ m, 40°) and user $\left(r_{2,1},\;\theta_{2,1}\right)=(3200$ m, 40°) effectively receive their respective signals without interfering with each other.

**Figure 7:**
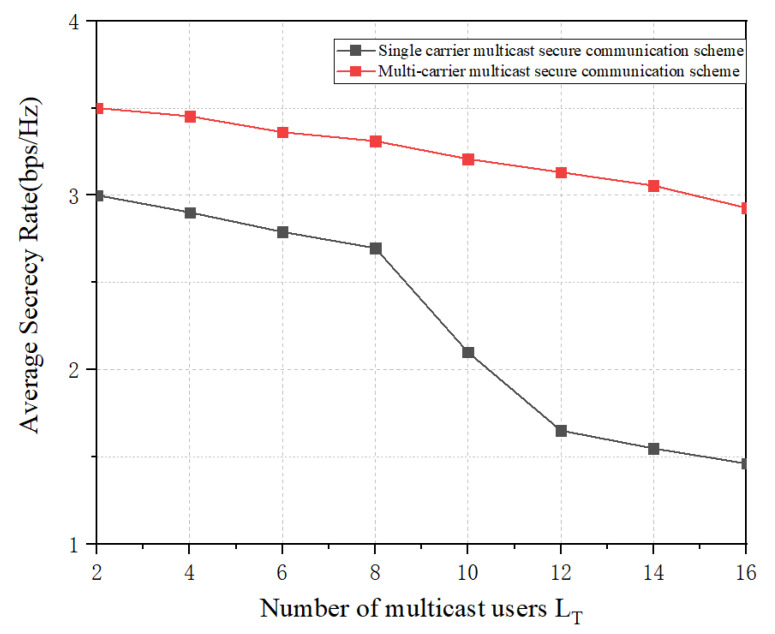
The relationship between the average security capacity of the system and the number of multicast users.

Figure 7 analyzes the relationship between the system security capacity of the proposed multi-carrier secure transmission method and the number of multicast users and compares it with the single-carrier secure transmission method. As can be seen from the figure, both the traditional single-carrier secure transmission method and the proposed method can provide good secure communication when fewer multicast users are expected. It is noteworthy that at this time, the average secure capacity of the proposed scheme is better than that of the single-carrier secure transmission method. This is mainly due to the fact that the proposed method has higher degrees of freedom for preferences, and the optimization algorithm for maximizing the artificial noise transmit power is proposed in the transmission method. When the number of multicast users increases, the security performance of the conventional single-carrier multicast group transmission method gradually decreases, and the difference in the average security capacity with the proposed method rapidly widens.

**Figure 8:**
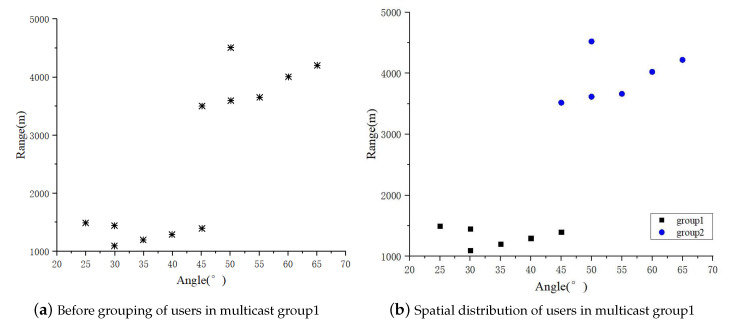
Spatial distribution of users in multicast group1.

**Figure 9:**
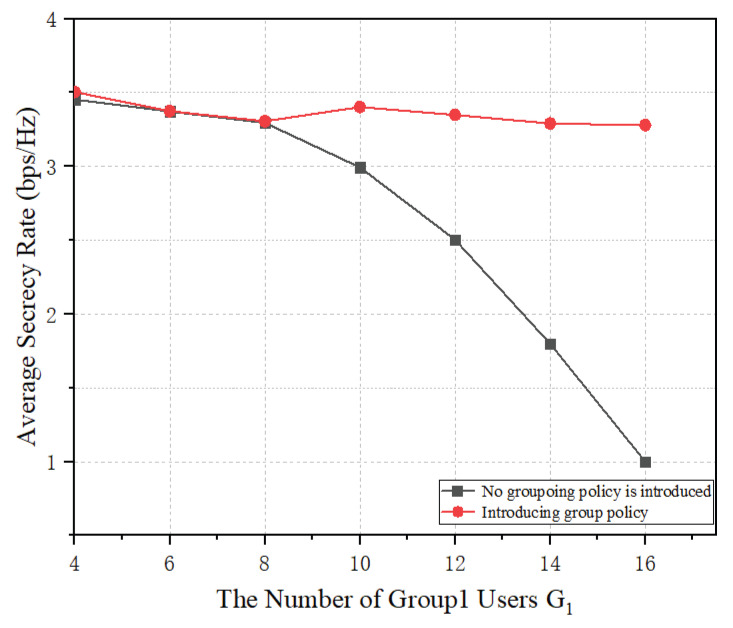
The relationship between the average security capacity and the number of users.

Next, Figure 8 as well as Figure 9 verify the feasibility of the multicast grouping strategy in terms of the number of frequency-controlled array transmitter antennas N=8, the number of available carriers per transmitter antenna as J=3, the number of multicast groups Q=2, the total number of multicast users $L_{T}=15$, and the number of users in multicast group1 as $G_{1}=12$. The spatial distribution of user locations in multicast1 is given in Figure 8a. Using the multicast grouping strategy, the users in multicast group1 form two independent multicast groups as shown in Figure 8b. Observation of the number of users and spatial locations after grouping shows that the number of users in each multicast group after regrouping is less than the number of transmitting antennas, and the multicast grouping strategy is able to classify the desired users with similar channels into a group, which is favorable for beam formation.

Figure 9 analyzes the relationship between the average security capacity and the number of users for multicast group1. From the figure, it can be seen that for multicast group1, after the introduction of the multicast grouping strategy, the users in multicast group1 are regrouped, and the information can be subsequently transmitted by using efficient beamforming and artificial noise techniques, which improves the security performance of the system. Especially when the number of users in the multicast group1 is larger than the number of transmitting antennas, the multi-carrier communication method without a multicast grouping strategy cannot ensure the secure communication of multicast users, and the average security capacity of the system decreases rapidly.

## 7. Conclusions

In the paper, a multi-carrier frequency-controlled array large-scale multicast group physical layer secure transmission method is proposed. First, the multi-carrier frequency-controlled array transmitter is designed to construct a large-scale multicast group system model. Second, the array weighting vector is optimized hierarchically with the objective of maximizing the artificial noise interference power while ensuring reliable signal reception by users, and the optimal solution is obtained using singular value decomposition and the Lagrange multiplier algorithm. Finally, through a large number of numerical simulations, it is shown that the multi-carrier large-scale multicast group secure transmission method realizes the secure and reliable communication of confidential information under a large-scale communication system, and the users in each multicast group can effectively receive the information while the eavesdroppers are inhibited from intercepting the confidential information to the maximum extent. Compared with the traditional single-carrier secure transmission method, the proposed method can ensure more users communicate securely. In the future, our research focus will be mainly on the integration of the FDA with real life and further research on practical applications related to the FDA.

## Data Availability

Data are available on request due to privacy/ethical restrictions.

## References

[1] Bari N., Mani G., Berkovich S. Internet of things as a methodological concept. Proceedings of the 2013 Fourth International Conference on Computing for Geospatial Research and Application.

[2] Shu F., Lu Z., Lin J., Sun L., Zhou X., Liu T., Zhang S., Cai W., Lu J., Wang J. (2019). Alternating iterative secure structure between beamforming and power allocation for UAV-aided directional modulation networks. Phys. Commun..

[3] Alam K.M., Saini M., El Saddik A. (2015). Toward social internet of vehicles: Concept, architecture, and applications. IEEE Access.

[4] Zou Y., Zhu J., Wang X., Hanzo L. (2016). A survey on wireless security: Technical challenges, recent advances, and future trends. Proc. IEEE.

[5] Sun X., Yang W., Cai Y., Tao L., Liu Y., Huang Y. (2018). Secure transmissions in wireless information and power transfer millimeter-wave ultra-dense networks. IEEE Trans. Inf. Forensics Secur..

[6] Huo Y., Fan X., Ma L., Cheng X., Tian Z., Chen D. (2019). Secure communications in tiered 5G wireless networks with cooperative jamming. IEEE Trans. Wirel. Commun..

[7] Stallings W. (2006). Cryptography and Network Security, 4/E.

[8] Wei Q., Wang B., Cao K. (2022). Physical Layer Security Technology Based on Nonorthogonal Multiple Access Communication. Mob. Inf. Syst..

[9] Tosi L., Rocca P., Anselmi N., Massa A. (2023). Array-Antenna Power-Pattern Analysis Through Quantum Computing. IEEE Trans. Antennas Propag..

[10] Gao J., Yuan Z., Qiu B., Zhou J. (2020). Secure multiusers directional modulation scheme based on random frequency diverse arrays in broadcasting systems. Secur. Commun. Netw..

[11] Cheng Q., Fusco V., Zhu J., Wang S., Wang F. (2019). WFRFT-aided power-efficient multi-beam directional modulation schemes based on frequency diverse array. IEEE Trans. Wirel. Commun..

[12] Qiu B., Tao M., Wang L., Xie J., Wang Y. (2019). Multi-beam directional modulation synthesis scheme based on frequency diverse array. IEEE Trans. Inf. Forensics Secur..

[13] Gao J., Yuan Z., Zhou J., Qiu B. (2020). Artificial-noise-aided energy-efficient secure multibeam wireless communication schemes based on frequency diverse array. Wirel. Commun. Mob. Comput..

[14] Chen L. Layered Security Multicast Algorithm based on Security Energy Efficiency Maximization in SCMA Networks. Proceedings of the 2021 7th International Conference on Computer and Communications (ICCC).

[15] Zhu B., Ge J., Huang Y., Yang Y., Lin M. (2014). Rank-two beamformed secure multicasting for wireless information and power transfer. IEEE Signal Process. Lett..

[16] Ju Y., Cao Z., Chen Y., Liu L., Pei Q., Mumtaz S., Dong M., Guizani M. (2023). NOMA-Assisted Secure Offloading for Vehicular Edge Computing Networks With Asynchronous Deep Reinforcement Learning. IEEE Trans. Intell. Transp. Syst..

[17] Diao D., Wang B., Cao K., Dong R., Cheng T. (2022). Enhancing reliability and security of UAV-enabled NOMA communications with power allocation and aerial jamming. IEEE Trans. Veh. Technol..

[18] Chen B., Li R., Ning Q., Lin K., Han C., Leung V.C. (2022). Security at physical layer in NOMA relaying networks with cooperative jamming. IEEE Trans. Veh. Technol..

[19] Baddi Y., Sebbar A., Zkik K., Maleh Y., Bensalah F., Boulmalf M. (2023). MSDN-IoT multicast group communication in IoT based on software defined networking. J. Reliab. Intell. Environ..

